# Human African Trypanosomiasis in South Sudan: How Can We Prevent a New Epidemic?

**DOI:** 10.1371/journal.pntd.0001541

**Published:** 2012-05-29

**Authors:** José A. Ruiz-Postigo, José R. Franco, Mounir Lado, Pere P. Simarro

**Affiliations:** 1 World Health Organization, Regional Office for the Eastern Mediterranean, Cairo, Egypt; 2 World Health Organization, Geneva, Switzerland; 3 Ministry of Health, Juba, Republic of South Sudan; Foundation for Innovative New Diagnostics (FIND), Switzerland

## Abstract

Human African trypanosomiasis (HAT) has been a major public health problem in South Sudan for the last century. Recurrent outbreaks with a repetitive pattern of responding-scaling down activities have been observed. Control measures for outbreak response were reduced when the prevalence decreased and/or socio-political crisis erupted, leading to a new increase in the number of cases. This paper aims to raise international awareness of the threat of another outbreak of sleeping sickness in South Sudan. It is a review of the available data, interventions over time, and current reports on the status of HAT in South Sudan. Since 2006, control interventions and treatments providing services for sleeping sickness have been reduced. Access to HAT diagnosis and treatment has been considerably diminished. The current status of control activities for HAT in South Sudan could lead to a new outbreak of the disease unless 1) the remaining competent personnel are used to train younger staff to resume surveillance and treatment in the centers where HAT activities have stopped, and 2) control of HAT continues to be given priority even when the number of cases has been substantially reduced. Failure to implement an effective and sustainable system for HAT control and surveillance will increase the risk of a new epidemic. That would cause considerable suffering for the affected population and would be an impediment to the socioeconomic development of South Sudan.

## Introduction

Human African trypanosomiasis (HAT), also known as sleeping sickness, is a deadly disease caused by subspecies of *Trypanosoma brucei* (Protozoa, Kinetoplastida)—*T.b. gambiense* and *T.b. rhodesiense*—transmitted to humans through the bite of insect vectors of the genus *Glossina* (tsetse flies) [1]–[5].

The disease has been a major public health problem in South Sudan for the last century [6]. Foci due to *T.b. gambiense* have been described in the Greater Equatoria Region bordering the Central African Republic [7], Democratic Republic of the Congo [8], [9], and Uganda [10], [11]. HAT caused by *T.b. rhodesiense* has been reported from areas of Jonglei state (Akobo County) bordering Gambella in Ethiopia [12], [13], although since 1984 no HAT cases have been reported from either Gambella or Jonglei [14].

In South Sudan, the main vector of the disease is *Glossina fuscipes*, but *G. tachinoides*, *G. pallidipes*, and *G. morsitans* have also been found in the Greater Equatoria Region [15]–[18].

Since the disease was first described in South Sudan, recurrent outbreaks with a repetitive pattern of response-scaling-down activities have been observed. Control measures for outbreak response were reduced when the prevalence decreased and/or socio-political crisis erupted, leading to a resurgence in the number of cases. That pattern may now reoccur due to difficulties in maintaining a high level of HAT control activities following the recent decrease in prevalence. Decision makers should call for urgent action to continue surveillance in order to avoid repeating that pattern. This is essential to achieve sustainable control of the disease.

South Sudan became a new nation in July 2011. The health services are still in the building phase and so far, insecurity is posing difficulties for health authorities and other implementers to easily access some HAT-endemic areas.

This paper reviews the available data and the various interventions over time, and reports on the current status of the disease.

The authors seek to raise international awareness of the threat of another sleeping sickness outbreak in South Sudan. To avoid this, an innovative disease control and surveillance approach needs to be developed. Failure to do so will inevitably result in a flare up of the disease, thus causing unnecessary suffering and significant interference with the socioeconomic development of South Sudan.

## Overview

### 1908–2000

**Figure 1:**
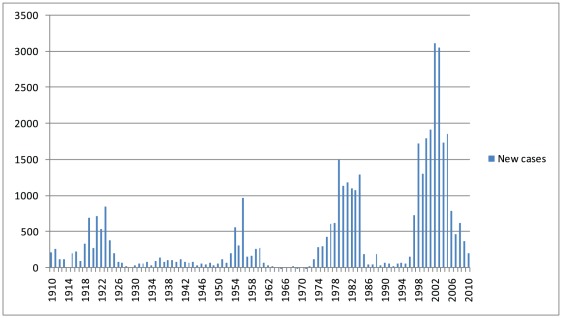
New cases of HAT reported in Sudan. Period 1910–2010.

Sleeping sickness was first reported from South Sudan in 1908 [19], [20]. Between 1920 and 1925, more than 3,000 cases were documented [21] (Figure 1). Control activities implemented from 1920 to 1950 brought the disease under control [22]. The civil war that began in 1955 resulted in the interruption of HAT control activities and a massive increase of the disease in the 1970s influenced by a high influx of returnees and refugees from HAT-endemic areas in Uganda. In 1974, this problem prompted the Government of Sudan to ask the World Health Organization (WHO) to assess HAT status. Active foci were identified and control measures proposed. At that time, the German Caritas Hospital in Nzara was the only center for sleeping sickness treatment [23], [24]. The joint Sudanese-Belgian Trypanosomiasis Control Program was set up in 1978 and achieved disease control by the mid-1980s. Li-Rangu and Juba became reference centers for HAT. Unfortunately, this program collapsed when the second civil war broke out in 1983. Predictably, HAT prevalence increased quickly, with the number of cases reported being higher than ever before [25]–[28].

### 2001–2005 and 2006–2010

From the late 1990s to early 2000s, several non-governmental organizations (NGOs) (namely Médecins Sans Frontières [MSF], International Medical Corps [IMC], CARE International, Malteser International, Samaritan's Purse, and Medical Emergency Relief International [MERLIN]) separately initiated HAT control activities in South Sudan. These NGO programs provided access to good quality case management, and they had the means to carry out active screening, which is a key component for controlling HAT. However, the NGOs were using different diagnostic and treatment protocols and their databases were not always accessible by the health authorities.

**Figure 2:**
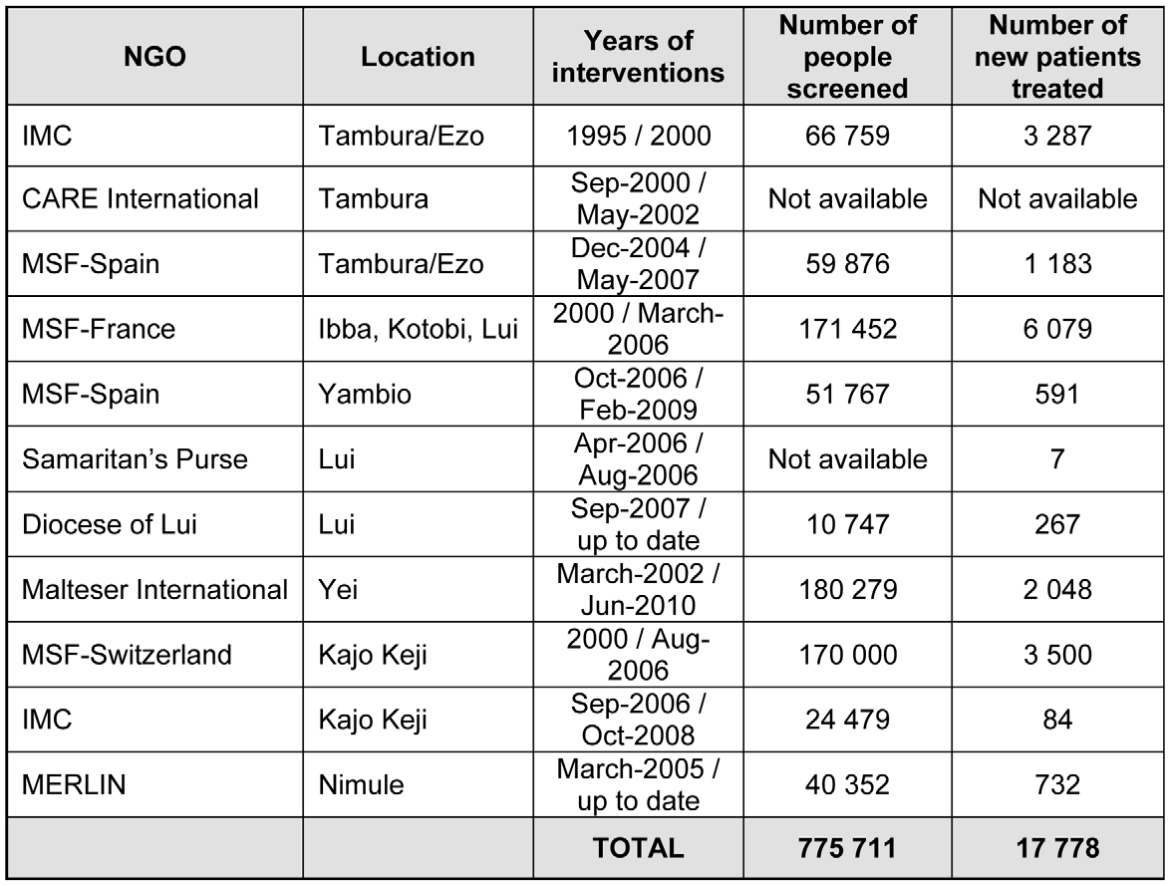
Number of people screened and patients treated according to NGO and period of intervention.

By 2005, there were 12 health facilities in Equatoria carrying out HAT control activities, 11 of them run by NGOs and one—in Juba—by the Ministry of Health (MoH). Active and passive screening and treatment implemented despite the social and armed conflict conditions under which they had to operate resulted in more than 500,000 people screened and more than 16,000 patients treated between 1998 and 2005 (Figure 2). This led to a significant decrease in the subsequent number of cases reported [29].

This decrease in prevalence resulted in most NGOs progressively stopping their activities after 2006, as it became difficult to advocate for internal and external resources for what no longer appeared to be a serious problem. By December 2010, only six health facilities were still carrying out HAT control (in Tambura, Yambio, Lui, Yei, Juba, and Nimule) and active screening had ceased completely. Four facilities were run by the MoH and two were run by NGOs (the Diocese of Lui, and MERLIN).

Since 2006, WHO has intensified its support for HAT control through provision of technical assistance, drugs and screening reagents, personnel training, and logistics.

**Figure 3:**
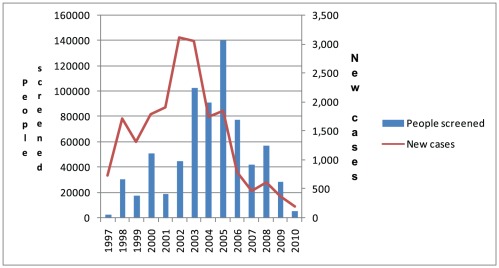
Number of people screened and cases reported of HAT in South Sudan. Period 1997–2010.

Comparison of control activity statistics during the 5 years following the departure of most NGOs (2006–2010) with the previous period of peak NGO presence (2001–2005) shows a 47% decrease in the number of people screened (from 399,977 to 211,946) and a 71% reduction in the number of new cases that were reported (from 8,664 to 2,475) (Figure 3). The percentage of screened people who underwent active screening was 70% in 2006 and fell to 8% in 2010. In 2006, 65% of new cases were in the second stage of the disease, while in 2010 that percentage increased to 76%.

## HAT Status by County 2000–2010

**Figure 4:**
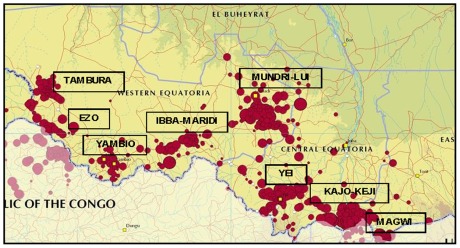
Areas reporting cases of HAT in South Sudan. Period 2000–2009.

Nine counties in Equatoria are endemic for HAT: Tambura, Ezo, Yambio, Maridi, Ibba, Mundri, Yei, Kajo Keji, and Magwi, representing a total population of 1.8 million according to the 2008 census (Figure 4). The 15,754 new cases reported from 692 locations between 2000 and 2009 have been mapped, and detailed epidemiological information on the disease was provided down to the village level [30]. Eight hospitals and five primary health care centers had the capacity for diagnosis and treatment at one time or another between 2000 and 2010, covering all the HAT-endemic counties.

### Tambura and Ezo Counties

From 1995 to 2000, IMC screened 66,759 people by active and passive case detection, and identified 3,287 new cases and 601 relapses. From September 2000 to May 2002, CARE International provided support only for passive screening and treatment. After May 2002, all activities ceased, treatment became unavailable, and in December 2003 the authorities of Tambura requested WHO assistance to deal with the sleeping sickness problem. WHO implemented a 6-month emergency program in 2004, offering passive screening, diagnosis, and treatment [31], with 9,354 people screened and 205 new cases and 29 relapses treated. WHO advocated for a longer term partner to engage in HAT control, and beginning in December 2004, MSF-Spain took over HAT control activities in Tambura and Ezo Counties. In mid-2007, MSF-Spain withdrew and HAT screening and treatment again became unavailable, so that patients could only get treatment by travelling to Yambio, some 200 km from Tambura. By mid-2010, the MoH resumed passive screening and treatment of patients at Tambura Hospital, and by the end of 2010, 234 people had been passively screened, with eight new cases and three relapses reported in Tambura.

### Yambio County

From 2000 to 2006, the hospitals of Yambio, Li Rangu, or Nzara had no capacity to provide diagnosis or treatment. HAT patients from Yambio County could only be diagnosed and treated in Ibba.

HAT diagnosis and treatment in Yambio Hospital were re-established by MSF-Spain in October 2006, and when MSF-Spain withdrew in February 2009, the MoH took over HAT services. Between 2006 and 2009, 51,767 people were screened and 591 new cases treated.

In 2010, 1,041 people were screened and 37 new cases reported. No relapses were reported.

### Mundri, Ibba, and Maridi Counties

In 2000, the MSF-Holland HAT control program in Ibba was handed over to MSF-France. Activities were progressively extended to Maridi and Kotobi hospitals in 2001 and Lui hospital in 2002. Over 170,000 people were screened and over 6,000 new cases treated between 1999 and early 2006 in these three counties [32]. The last active screening was carried out in Ibba and Maridi in 2004, where the overall prevalence found was below 1%, although in two locations—Baama and Maribindi—the prevalence was above 1%. In Mundri County, prevalence was higher than 1% in five locations (Lanyi, Amadi, Diko, Nyau, and Kalalai) in 2004 and 2005 when the last active screening was carried out by MSF-France.

When MSF-France withdrew from the area in March 2006, active case finding ceased: Ibba and Kotobi HAT centers were closed, and the Maridi and Lui hospitals stopped treatment for second-stage HAT. Later in 2006, both hospitals also discontinued treatment of first-stage disease. Patients had to travel some 200–300 km to seek HAT medical care either in Yambio, Juba, or Yei.

In 2008, a rapid epidemiological assessment was carried out by WHO and the Diocese of Lui in seven villages (Lozoh, Milikolongo, Mundri, Ndibo, Amadi, Osho, and Jambo) in Mundri County. The assessment showed a prevalence of 0.46% (eight cases out of 1,722 people screened). No active screening has been carried out in those villages since then.

As of today, Maridi hospital has neither resumed diagnosis nor treatment of sleeping sickness.

In September 2007, representatives of Lui hospital, managed by the Diocese of Lui, contacted WHO to request support to resume HAT medical services in their center. HAT diagnosis and treatment were made available, including treatment for second-stage patients for the first time in that hospital. From 2007 to 2009, over 9,000 people were passively screened in Lui hospital and 223 new cases and 113 relapses were treated, including 35 patients previously treated in Lui and 78 treated in other treatment centers. In Lui hospital in 2010, 1,406 people were screened and 44 new cases and 52 relapses (28 previously treated in Lui and 24 in other centers) reported. An in-depth analysis should be done to precisely identify the reasons behind that high relapse rate in Lui hospital.

### Kajo Keji County

When MSF-Switzerland initiated a HAT control program in 2000 in Kajo Keji County, they built a HAT hospital in Kiri. In 2004, when the overall prevalence found during active screening had decreased to 0.7%, HAT activities were moved to the Kajo Keji hospital, where 20 beds were allocated for the disease. In 2005, prevalence detected during active screening was 0.3% and MSF-Switzerland stopped its activities there in 2006. From 2000 to 2006, over 170,000 people were screened and around 3,500 cases were diagnosed and treated [33]–[35].

In September 2006, IMC took over HAT activities from MSF-Switzerland in Kajo Keji hospital. During 2007–2008, IMC screened 20,098 people and treated 64 new cases. IMC ceased HAT medical services in October 2008, and to date the County Health Department (CHD) has not resumed these activities. Since then, several cases coming from Kajo Keji have been diagnosed in Nimule, Yei, and Juba and even in bordering HAT centers in Uganda (Moyo hospital). No active screening has been performed in the county since 2008.

### Yei County (including Lainya and Morobo)

In 2000 and 2001, cases of HAT coming from Yei were diagnosed and treated in Ibba and Omugo (Uganda). A rapid epidemiological assessment performed by Malteser International with support of the WHO in 2001 provided estimates of HAT prevalence at around 4% to 7% in some highly endemic villages in Yei County. Malteser International started implementing HAT control activities in Yei in March 2002 at the St. Bakhita Health Centre on the premises of the Diocese of Yei. A specific ward and laboratory for HAT were set up. From 2002 until 2005, more than 130,000 people were screened and 1,592 new cases detected and treated. The prevalence observed in the screened population dropped from 4.03% in 2002 to 0.38% in 2005.

Between 2006 and 2009, 49,610 people were screened and 437 new cases reported. In mid-2010, Malteser International handed over the HAT control activities to Yei Civil Hospital run by the MoH.

In 2010, 1,771 people were passively screened, and 73 new cases and four relapses reported and treated.

### Juba County

In the late 1990s and early 2000s, occasional cases (3–6 per year) coming from internally displaced people or military camps were diagnosed in Juba Teaching Hospital (JTH). In 2002, an active screening survey carried out by the Tropical Medicine Research Institute-TMRI (Khartoum) on 2,344 individuals in 13 locations showed a CATT whole blood seroprevalence of 11% but no serological suspect was parasitologically confirmed. In 2003, a joint WHO/TMRI active screening survey in six locations, including internally displaced people camps, showed a seroprevalence of 4% and a parasitological prevalence of 0.13% (3/2,374).

Between 2006 and 2009, 450 people were screened at JTH and 90 new cases reported, most coming from Mundri County.

In 2010, 18 people were screened and nine new cases reported, mainly coming from neighboring counties.

### Magwi County

Between 2000 and 2005, sporadic cases from Magwi County were diagnosed and treated in Kajo Keji. In March 2005, MERLIN started HAT control activities in Magwi County, and a rapid assessment in 16 villages showed an overall prevalence of 1% (25/2,481).

MERLIN continues to provide support to all medical services at Nimule hospital including HAT. From 2005 to 2009, 39,043 people were screened and 703 new cases reported.

In 2010, 1309 people were screened, with 29 new cases and six relapses reported.

## Case Management

South Sudan was the first place where eflornithine was used on a large scale as first line treatment for second-stage HAT patients. The decision was made due to melarsoprol toxicity [36]–[38] and an increased relapse rate observed with melarsoprol in some areas of South Sudan [39]–[40] and neighbouring foci in Uganda [41]. Treatment with eflornithine was found to be safer [42], and melarsoprol treatment was progressively replaced by eflornithine as first line treatment for second-stage HAT.

Eflornithine treatment was introduced in 2001 by MSF-France in Maridi [43], [44] (Ibba and Kotobbi hospitals), in 2002 by Malteser in Yei [45], [46], in 2004 by MSF-Switzerland in Kajo-Keji [47], in 2004 by the WHO-supported treatment center in Tambura, in 2005 by MERLIN in Magwi [48], in 2007 by the Diocese of Lui, and in 2010 by the MoH in Juba. All drugs to treat HAT were made available free of charge by WHO through public/private agreements between WHO and the manufacturers [49].

Data on eflornithine effectiveness in 944 new cases treated in the period 2002–2005 was reviewed by WHO in collaboration with NGO partners from Yei and Kajo Keji hospitals showing a 9% (8%–11%) relapse rate (unpublished data).

In mid-2010, nifurtimox-eflornithine combination therapy (NECT) [50] was introduced as the new first line for second-stage treatment in South Sudan, shortly after the inclusion of that treatment regimen in the 2009 WHO Model List of Essential Medicines [51]. A training of trainers was organized by WHO for key staff in all HAT centers.

## Vector Control

HAT control activities in South Sudan focused on active and passive case detection and prompt treatment of cases. Some short-term vector control activities were implemented in selected areas by Malteser International in Yei and CARE International in Tambura [52].

## Monitoring and Surveillance

Coordination between the MoH, WHO, and NGOs took place from 2003 to 2008 through HAT annual review meetings held to discuss achievements and challenges in the control of the disease. These meetings were discontinued in mid-2008. A standardized HAT monthly reporting form was created in 2007 and became part of the Integrated Disease Surveillance and Response information system. Despite this reporting system, obtaining regular data at the central level remains a major challenge.

## Institutional Framework

HAT control and surveillance are within the mandate of the Director General of Community and Public Health in the MoH–Republic of South Sudan (RSS) [53]. The MoH-RSS is responsible for policy making, technical guidance, and overall coordination, whereas implementation of control activities is the responsibility of the State Ministries of Health (SMoH) and the CHDs. Three SMoH are involved in the implementation of HAT activities—Eastern, Central, and Western Equatoria—but these ministries and departments have so far been unable to take over the control activities carried out in the past by NGOs. The substantial decrease in the number of people screened reflects this weakness of the health system.

## Conclusions

From the mid-1990s until 2005, large-scale HAT control activities in South Sudan were carried out by NGOs, leading to an important decrease in the number of cases that caused a progressive withdrawal of NGOs involved in HAT control. From 2006 onward, control interventions and treatment centers providing services for sleeping sickness have been reduced, and population access to HAT diagnosis and treatment has been considerably diminished. Epidemiological history shows that such a situation could lead to a new epidemic, and it may be that an unreported resurgence of disease is already occurring. That idea is supported by the 2010 figures, where 92% of the screening was done passively and 76% of patients were diagnosed as already with second-stage disease. Since most HAT first-stage patients are asymptomatic, or present with symptoms mimicking other common diseases such as malaria, they are usually diagnosed in higher numbers through active screening. Meanwhile, those actually infected and not being treated act as a reservoir, maintaining the transmission cycle.

The risk of a new epidemic is aggravated by four facts:

1. Reduced HAT control activities result in diminishing local capacity for the control of sleeping sickness, unless the remaining competence is used to train younger staff to resume HAT surveillance and control activities.
2. The directorate responsible for HAT control is also in charge of other communicable diseases and reporting recurrent outbreaks, such as cholera, meningitis, and visceral leishmaniasis. As a result, it becomes difficult to keep HAT, with a less “visible” progression, on the top of the agenda at a time when the number of cases has been substantially reduced and the disease is thus not regarded as a major public health problem.
3. Most NGOs who have been active in HAT control are focused on providing medical aid in emergency situations. The situation of HAT does not currently qualify as a public health emergency.
4. The health system of South Sudan is not yet consolidated and consequently not able to fully take over the control activities carried out in the past by NGOs. Having been unsuccessful in convincing most NGOs to continue their control activities in the region, the WHO is now strengthening SMOH and CHD capacity to allow them to fill this role.

Strengthening current HAT control and surveillance capacity and activities includes the following:

i) reinforcing selected health care facilities to deliver HAT diagnosis and treatment; ii) improving the HAT surveillance system by ensuring passive screening and reporting to the central level; iii) reinforcing central level for management and data analysis; and iv) establishment of a national team that can respond to the possible re-emergence of the disease, as deduced from the data from passive screening with targeted active case search in the affected areas.

Failure to implement an effective and sustainable system for HAT control and surveillance will pose a high risk of HAT resurgence, which would cause immeasurable suffering for the affected population and would be an impediment to the socioeconomic development of South Sudan.

**Key Learning Points**

- Recurrent outbreaks of sleeping sickness with a repetitive pattern of response-scaling-down activities have been observed. Control measures for outbreak response were reduced when the prevalence decreased and/or socio-political crisis erupted, leading to a resurgence in the number of cases.
- Sleeping sickness, with a less “visible” progression, is very difficult to keep on the top of the agenda at a time when the number of cases has been substantially reduced, and the disease is thus not regarded as a major public health problem.
- Failure to implement an effective and sustainable system for human African trypanosomiasis control and surveillance will increase the risk of a new epidemic. That would cause considerable suffering for the affected population and would be an impediment to the socioeconomic development of South Sudan.

**Key Papers**

- Smith DH, Pepin J, Stich AH (1998) Human African trypanosomiasis: an emerging public health crisis. Br Med Bull 54(2): 341–355.
- Moore A, Richer M (2001) Re-emergence of epidemic sleeping sickness in southern Sudan. Trop Med Int Health 6: 342–347.
- Malvy D, Chappuis F (2011) Sleeping sickness. Clin Microbiol Infect 17(7): 986–995.
- Brun R, Blum J, Chappuis F, Burri C (2010) Human African trypanosomiasis. Lancet 375(9709): 148–159.
- Simarro PP, Cecchi G, Paone M, Franco JR, Diarra A, et al. (2010) The atlas of human African trypanosomiasis: a contribution to global mapping of neglected tropical diseases. Int J Health Geogr 9(1): 57.
